# Inductive Textile Sensor Design and Validation for a Wearable Monitoring Device

**DOI:** 10.3390/s21010225

**Published:** 2021-01-01

**Authors:** Astrid García Patiño, Carlo Menon

**Affiliations:** Menrva Research Group, Schools of Mechatronic Systems & Engineering Science, Simon Fraser University, Metro Vancouver, BC V5A 1S6, Canada; agarciap@sfu.ca

**Keywords:** inductance, textile sensors, wearable device, smart garment, E-textiles

## Abstract

Textile sensors have gained attention for wearable devices, in which the most popular are the resistive textile sensor. However, these sensors present high hysteresis and a drift when stretched for long periods of time. Inductive textile sensors have been commonly used as antennas and plethysmographs, and their applications have been extended to measure heartbeat, wireless data transmission, and motion and gesture capturing systems. Inductive textile sensors have shown high reliability, stable readings, low production cost, and an easy manufacturing process. This paper presents the design and validation of an inductive strain textile sensor. The anthropometric dimensions of a healthy participant were used to define the maximum dimensions of the inductive textile sensor. The design of the inductive sensor was studied through theoretical calculations and simulations. Parameters such as height, width, area, perimeter, and number of complete loops were considered to calculate and evaluate the inductance value.

## 1. Introduction

Electronic textiles, also known as E-textiles or smart garments, could be a solution for monitoring daily activities due to their small size, light weight, and simple operation [1,2]. As a result, they can comfortably be worn by participants without obstructing their daily activities.

Inductive textile sensors are made from highly conductive materials (e.g., copper wire, stainless steel yarn, conductive threads). These sensors base their working principle on their capacity to create a magnetic field when an electrical current is passed through loop(s) of the conductive threads [3]. The sensor’s output is modified when the shape of the magnetic field changes. These changes are the result of deforming the sensor due to an externally applied force. Fava et al. [4] reported that the inductance and the sensitivity (Δ Inductance/Δ strain) of the sensor can be increased by augmenting the number of coils and/or narrowing the width and space between the coils [4]. Typically, inductive sensors are manufactured with a circular coil shape, however, they are not limited to only this shape [5]. The versatility of the inductive textile sensors enables the ability to embed or affixed these sensors to different surfaces. Inductive sensors are regularly used in antennas [6,7] and plethysmographs [5,6,8].

Yoo [9] and Coosemans et al. [10] used inductive-type sensors for wireless-powered applications. Coosemans et al. [10] created a platform using these types of sensors to transmit ECG measurement data. To measure the heartbeat, Koo et al. [11] developed a magnetic-induced conductivity sensing module shaped in a coil configuration using nine strands of silver-polyester hybrid yarn. Wijesiriwardana [12] manufactured a knitted sensor made with Lycra and copper wire to measure strain and displacement, suggesting the possibility of expanding the sensor’s applications to respiratory measuring and motion and gesture capturing systems. This sensor was reported to be ideal for wearable devices given its unobtrusive behavior, small size, lightweight, comfort, and tightfitting properties. Wu et al. [13] presented a wearable inductive plethysmography to monitor respiration during sleep. This inductive plethysmography showed high reliability and low production cost.

Commonly, monitoring E-textile devices are based on resistive sensors. However, resistive sensors present important disadvantages that limit their practicality, such as high hysteresis, non-linearity of their response, and a drift in their readings when a certain amount of stretch is held for a period of time [2]. The aforementioned disadvantages can be overcome through the use of inductive textile sensors since these sensors do not present a drift in their output signal over time, which makes them a reliable monitoring system for an extended period of time. Additionally, inductive textile sensors’ output signals present minimal noise, almost linear behavior, almost no hysteresis, straightforward signal processing, and do not require specialized equipment or materials [2,14,15]. Unfortunately, there is little information regarding the design and manufacture of the inductive textile sensors.

In this study, the process for designing an inductive strain textile sensor with a flat rectangular coil configuration is proposed. The inductance value behavior was studied based on the change of its dimensions and number of complete loops. A series of simulations were performed to validate the theoretical calculations and evaluate the inductance behavior when variables such as the material of the sensor and its surrounding was considered. Finally, the design process was applied to design and develop an inductive textile sensor to monitor the trunk forward bending [3].

## 2. Design Process

**Figure 1:**
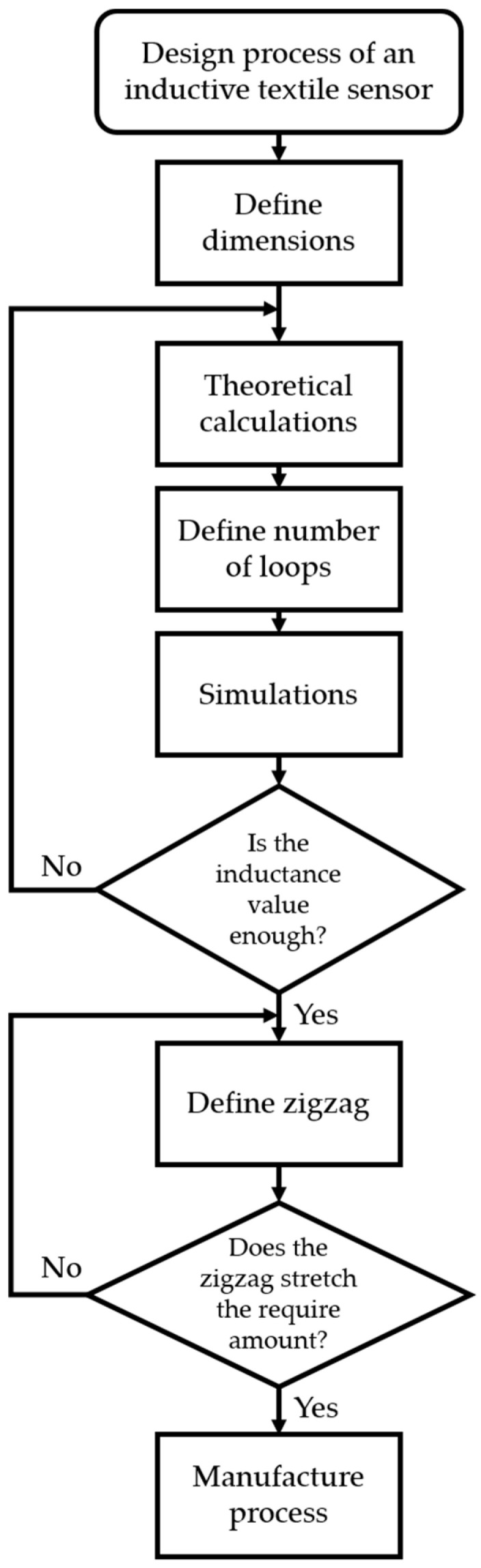
Diagram for the design process of an inductive strain textile sensor with a flat rectangular coil configuration.

In this study, we investigated the design and validation of an inductive strain textile sensor. The design of the sensor started by defining its size. Then, the inductance value of the sensor was theoretically calculated using equations from the literature to understand the behavior of the inductance when a change in the geometry of the sensor occurred. This was followed by a comparison between different theoretical calculations based on perimeter/area and height/width of a single loop rectangle. Additionally, theoretical calculations based on the number of complete loops of a flat rectangular coil were investigated. Next, a series of simulations were investigated to verify the values obtained from theoretical calculation and the impact of including variables such as the material of the sensor and its surrounding environment. Figure 1 illustrates the design process for the inductive strain textile sensor.

### 2.1. Defining Maximum Size

The goal of this step was to numerically identify the total size where the sensor was going to be placed. In the case of wearable textile sensors, its size can be defined by the anthropometry. Anthropometry is the human science that studies body measurements such as body size, shape, strength, and working capacity [16]. Investigation into the anthropometrics of the target region is recommended.

### 2.2. Theoretical Calculation of the Inductance Value for a Flat Rectangular Coil Sensor

Two approaches for the designing of the sensor were considered: First, the calculation of a simple rectangle based on its dimensions, such as height, width, perimeter, and area [17,18,19], and second, the calculation of a flat rectangle coil using the Terman equation was performed [18]. In both approaches, the inductance behavior was analyzed when the height, width, perimeter, area, or number of loops were modified.

#### 2.2.1. Inductance of a Rectangle with Round Wire

Thompson [17] and Grover [19] presented several equations to calculate the inductance based on the shape of an antenna and the type of wire used. The two equations used to calculate the inductance of a rectangle are [17,19]:

(1) $$L≃\;\frac{\mu_{0}p}{2\pi }\left[\ln\left(\frac{2p}{R}\right)+0.25-\ln\left(\frac{p^{2}}{a}\right)\right]$$

(2) $$\begin{array}{ll} L≃\frac{\mu_{0}\mu_{r}}{\pi }[-2(W & +H)+2\sqrt{H^{2}+W^{2}}-H\ln\left(\frac{H+\sqrt{H^{2}+W^{2}}}{W}\right)\\ & -W\ln\left(\frac{W+\sqrt{H^{2}+W^{2}}}{H}\right)+H\ln\left(\frac{2H}{R}\right)+W\ln\left(\frac{2W}{R}\right)]\; \end{array}$$

where *µ*_0_ is the magnetic permeability of free space equal to 4п × 10^−7^ H/m, and *µ*_r_ is the relative permeability of the material inside the rectangle loop. The variable *µ*_r_ is considered to be air, the value of which is 1. The perimeter of the polygon is *p*, the area of the polygon is *a*, the width of the rectangle is *W*, the height of the rectangle is *H*, and finally, the radius of the wire is *R*.

**Figure 2:**
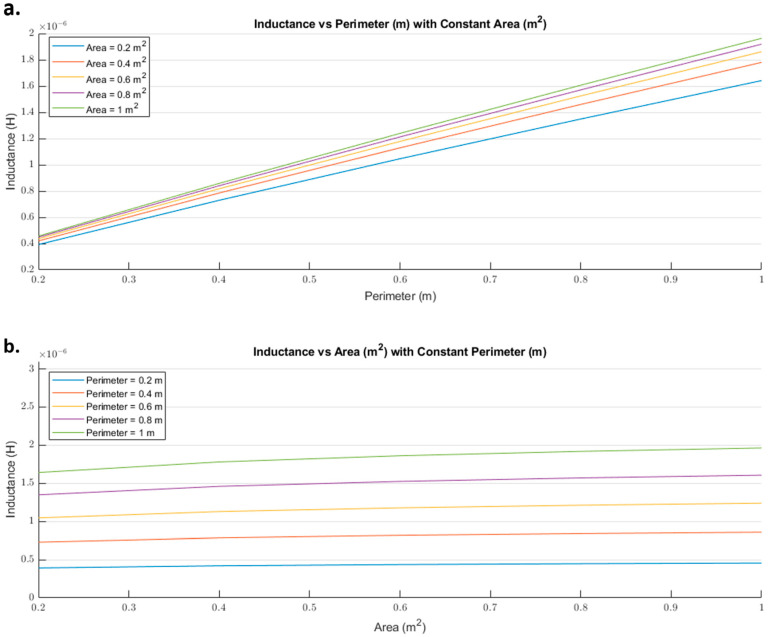
Inductance (H) behavior based on the area (m^2^) and perimeter (m) of a polygon using a round wire: (**a**) Inductance value vs. perimeter with a constant area; (**b**) inductance vs. area with a constant perimeter.

Equation (1) calculates the inductance of a polygon, with any perimeter and area, composed of a round wire. Figure 2 shows the inductance behavior based on Equation (1). From Figure 2, it was noticeable that the inductance increased with an almost linear behavior when the area was kept constant and the perimeter increases.

**Figure 3:**
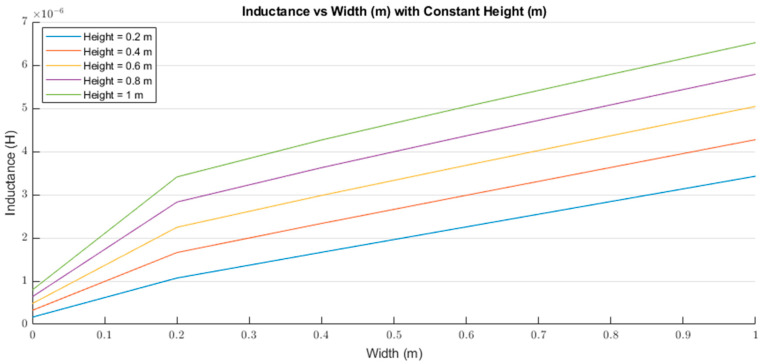
Inductance (H) behavior based on the height (m) and width (m) of a rectangle loop.

Equation (2) calculates the inductance value according to the height and width of the rectangle loop. Figure 3 illustrates the behavior of Equation (2), where both height and width are in meters and the inductance is in henries. From Figure 3, we observed that the inductance rapidly increased with a linear behavior when the height was kept constant and the width increased. Equation (2) shows a linear behavior regardless of the variable kept constant. The inductance value will rapidly decrease when either the width or the height is closer to zero due to the natural logarithms in Equation (2). Additionally, both equations neglected the loop’s material, but did consider the radius of the wire.

#### 2.2.2. Flat Rectangular Coil

**Figure 4:**
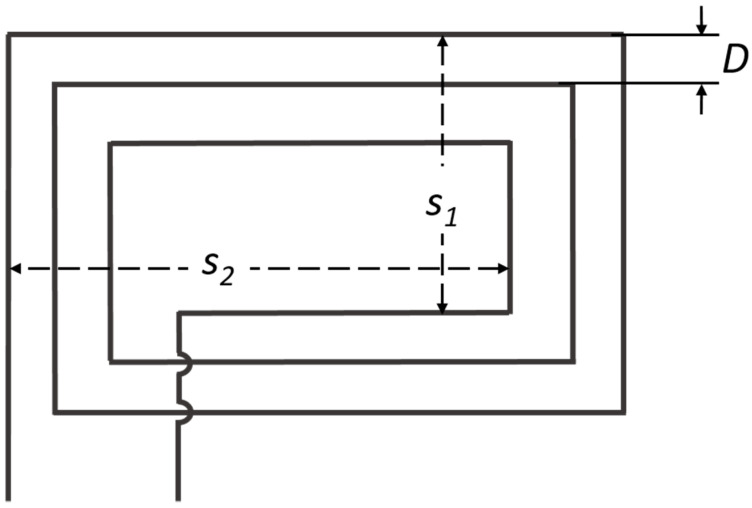
Flat rectangle coil geometry presented by Terman. Adapted from [18].

Terman [18] developed Equation (3) to calculate the low-frequency inductance of a flat rectangular coil. This equation depends on the average dimensions of the rectangle and the number of complete turns of the wire [18]:

(3) $$\begin{array}{ll} L≃\;0.02339n^{2} & \left[\left(s_{1}+s_{2}\right)log_{10}\frac{2s_{1}s_{2}}{nD}-s_{1}log_{10}\left(s_{1}+g\right)-s_{2}log_{10}\left(s_{2}+g\right)\right]\\ & +0.01016n^{2}\left(2g-\frac{s_{1}+s_{2}}{2}+0.447nD\right)\\ & -0.01016n\left(s_{1}+s_{2}\right)\left(A+B\right)\; \end{array}$$

where *s*_1_ and *s*_2_ are the average dimensions of the rectangle, *g* is the average diagonal $g=\;\sqrt{s_{1}{}^{2}+s_{2}{}^{2}}$, *n* is the number of complete turns with a pitch of winding *D*. Figure 4 illustrates the flat rectangular coil configuration.

Furthermore, *A* and *B* are correction constants based on the wire spacing and the number of turns, respectively. Table 1 shows the correction constants for *A* from 0.01 to 0.1, and Table 2 shows the *B* correction constants from 1 to 10. Complete tables for correction constants *A* and *B* are found in the Radio Engineers’ Handbook by Terman [18]. Terman used the English system for calculations in Equation (3), therefore, the dimensions are in inches.

Equations (1)–(3) do not consider the material of the sensor. Moreover, the diameter of the wire is only considered in the correction constant *A*. The geometry and symmetry of the sensor in Equation (3) are extremely important given that average dimensions (*s*_1_, *s*_2,_ and *g*), as well as the distance between loops D, are considered. Therefore, a slight modification in the geometry of the sensor during the manufacturing process can have a great impact on the inductance value.

### 2.3. Simulations

In this study, all the simulations were performed in Ansys 2019 R2/19.4 Electromagnetics (Ansys Inc., Canonsburg, PA, USA) using Maxwell 3D design. The objective of the simulations was to validate the theoretical calculations and evaluate the change of inductance value when variables such as the material of the sensor differed.

**Figure 5:**
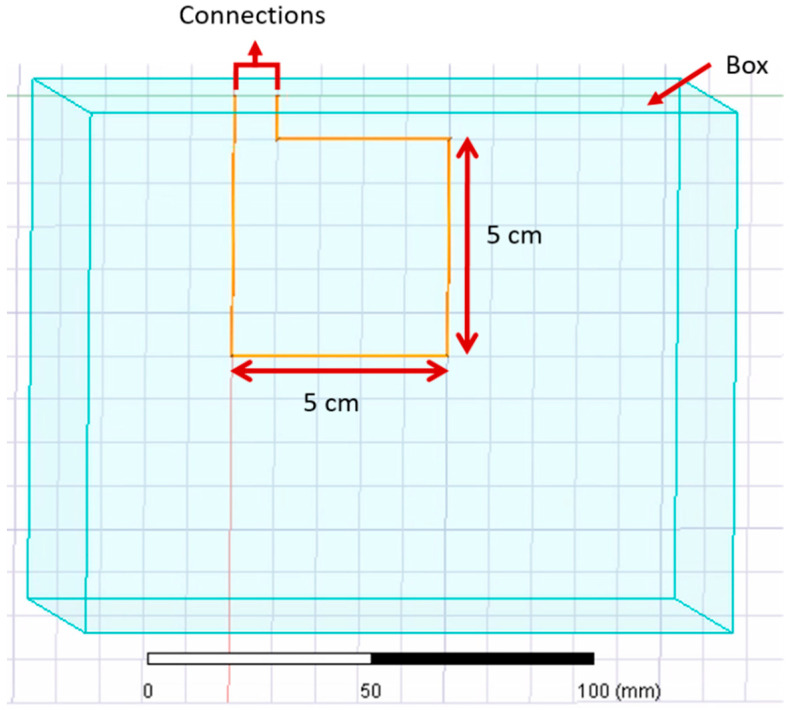
The sensor’s characteristics used to compare the simulated inductance value of different materials.

**Figure 6:**
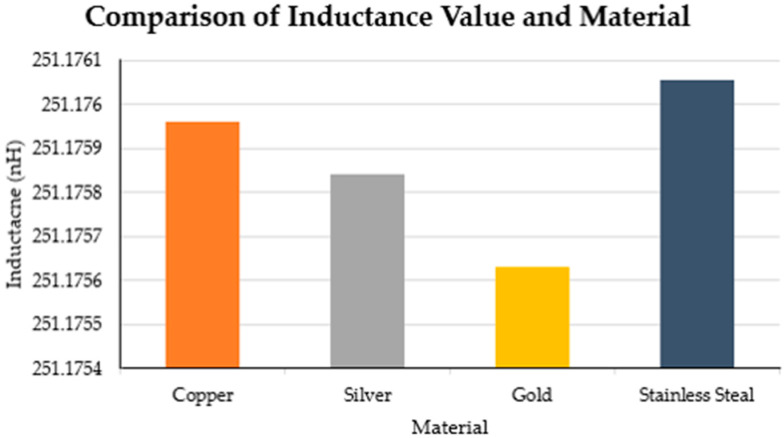
Comparison of simulated inductance value of the same sensor using copper, silver, gold, and stainless steel.

Previous studies used copper, silver, and stainless steel to manufacture inductive textile sensors for diverse applications [8,10,11,12,20,21]. We performed a series of simulations to evaluate the change of the inductance value when different materials were used for a single loop sensor. Table 3 shows the parameters used in the Ansys for the comparison of materials for the same inductive sensor. Figure 5 illustrates the characteristics of the simulated sensor used for this section. Finally, Figure 6 illustrates the comparison on the inductance value of a simulated inductive sensor using different materials such as copper, silver, gold, and stainless steel.

In Table 3, “Sensor’s Characteristics” describes the properties used for this comparison. The parameter “Box” describes the dimensions and material surrounding the inductive sensor (light blue).

The total change range of the inductance value obtained from using different materials was 0.424 pH. The material that obtained the higher inductance value was stainless steel with an inductance value of 251.1761 nH, followed by copper with an inductance value of 251.1760 nH. The inductance difference between both materials was 0.095 pH. The material that had the lowest inductance value was gold with an inductance value of 251.1756 nH. The effect of the material on the inductance value can be neglectable when the working range of the inductive sensor is in nH or higher.

### 2.4. Zigzag Properties

The zigzag pattern used in the design of the inductive sensor is a property that provided an increase of inductance of 35% when compared with a straight line [3]. Furthermore, the zigzag pattern allowed the sensor to be stretched without damaging it. In a previous study [3], we presented data that showed the width of the zigzag pattern had an effect on the inductance value. The inductance value increased when the width of the zigzag pattern was reduced. Moreover, zigzag widths of 2 and 4 mm were able to be stretched up to 200% of their original length.

### 2.5. Manufacture Process

The integration of inductive textile sensors into a garment or fabric can be done by sewing or knitting [3,8,10,11,12,14,20,21,22].

The sewing technique allows for the integration of textile sensors into the fabric during the manufacturing process. This advantage provides the possibility of selecting the best stitch for each type of fabric [23,24]. Some popular stitches used for stretchable fabrics are zigzag, curve, wave, and sinusoidal pattern. Sewing textile sensors into the fabric or garment presents several advantages such as geometry versatility, manufacturing ease, and the ability to replace the sensor without damaging the garment or fabric.

Using the knitting technique, textile sensors are created with a flat-bed knitting machine using either interlocking or plain knitted structures. These sensors have the advantage of conforming to the shape of the body, as well as improved elasticity and breathability [22,23]. This technique can be done with a variety of conductive yarns, such as copper, silver-coated nylon yarns, polyester-blended yarn with stainless steel fibers, and double covered elastomeric yarns [6,12,22,23,25].

## 3. Example Case

**Figure 7:**
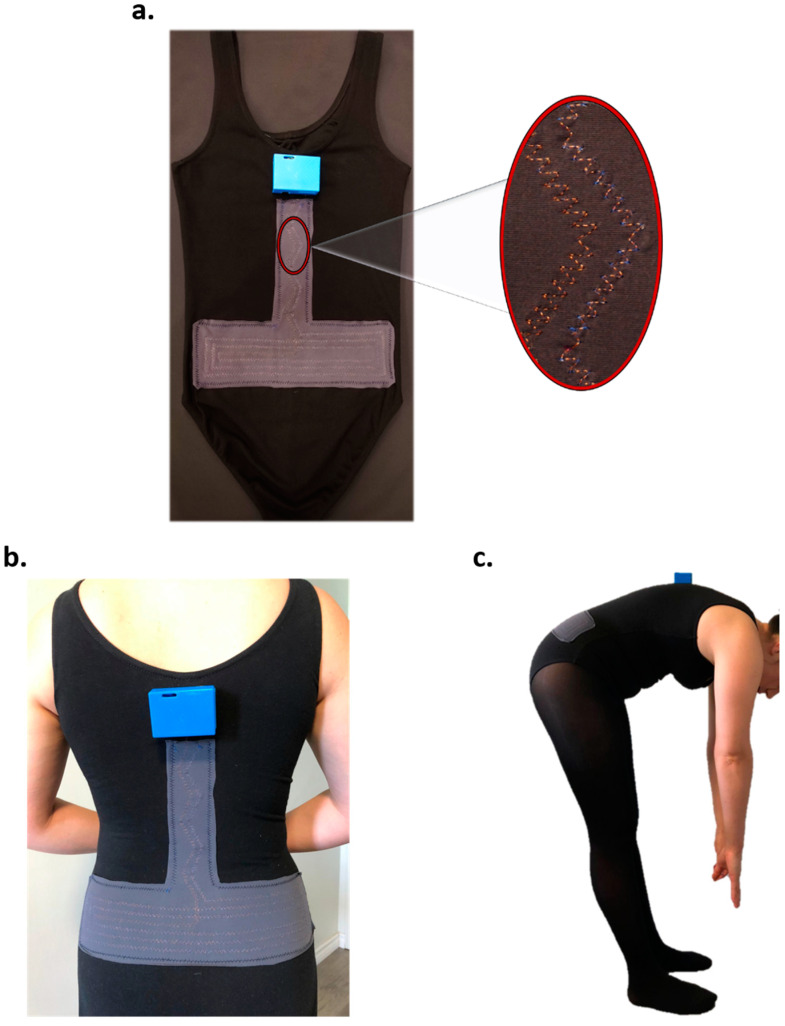
Smart garment: (**a**) Inductive strain textile sensor attached to a leotard [3]; (**b**) rear view of the smart garment worn by a participant; (**c**) participant performing forward bending while wearing the smart garment.

In this section, we present an example cause of an inductive strain textile sensor created following the presented methodology (Figure 1). The textile sensor was made by sewing a single copper wire with a diameter of 0.14 mm into a piece of elastic fabric. The wire diameter was selected based on its close similarity to the diameter of common thread and its possibility to be attached to the fabric using a sewing machine. Furthermore, the inductance value of the textile sensor increases with a smaller wire diameter. The detailed manufacture and performance of the aforementioned sensor was presented in [3]. Figure 7 illustrates the manufactured inductive sensor and the final prototype being worn by a participant.

### 3.1. Anthropometry

The objective of the sensor as designed was to detect forward bending of the trunk while rejecting other movements, such as lateral bending or twisting [3]. To achieve this goal, the configuration and placement of the sensor was chosen strategically. Previous studies reported that when an individual wearing a tight-fitting shirt bends forward, the lumbar section of the back undergoes major strain [26]. The trunk movements in the frontal and horizontal planes, which correspond to lateral bending and rotation, cause a smaller strain on this section [26]. According to this evidence, the inductive strain textile sensor was positioned on the lumbar area, using a flat rectangle coil shape.

Given that more than 90% of nurses are female [27,28,29], the anthropometry of a healthy female was used as the reference in designing and testing the inductive strain textile sensor developed in this study.

**Figure 8:**
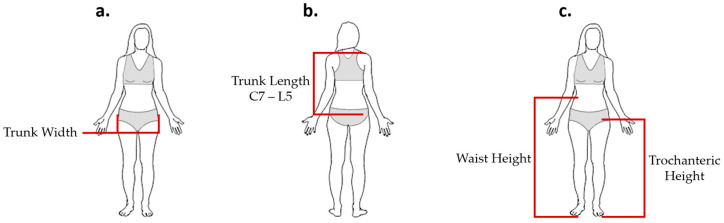
Anthropometric dimensions. (**a**) Trunk width at the iliac wrest [32], (**b**) trunk length from C7 to L5 [31], (**c**) waist height and trochanteric height [30]. Adapted from [34].

The trunk’s general anthropometry dimensions of a healthy female using a 95th percentile [30] and anthropometrics measurements gathered by previous studies [31,32,33] was employed in the design of the sensor. The collected measurements are summarized in Table 4 and illustrated in Figure 8.

**Figure 9:**
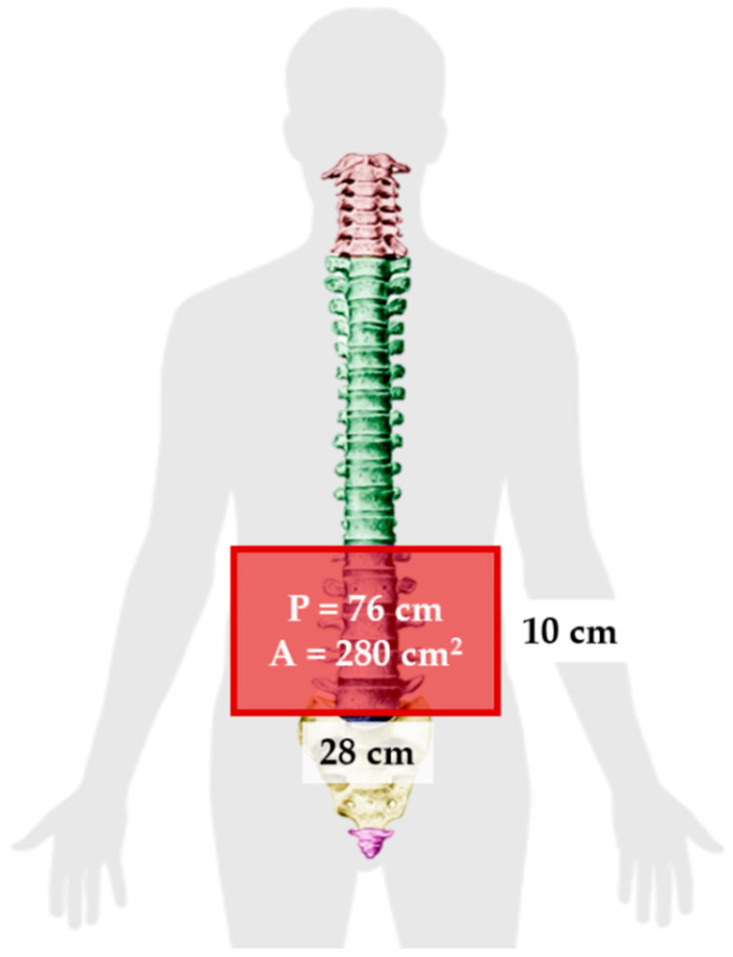
Maximum dimensions for the inductive sensor design. P and A represent the perimeter and the area, respectively. This image is licensed under a Creative Commons Attribution-Share Alike license (CC BY—SA) [35,36].

Podbevsêk [33] reported the distance between the waist and hip to be approximately 20 cm. On the other hand, the National Aeronautics and Space Administration (NASA) Anthropometry Source Book [30] reported that the distance between the trochanteric height and waist height was approximately 21 cm (shown in Table 1). Given these measurements, the total height from L1 to S5 was approximated to be 20 cm for a healthy female of 21–40 years old. We excluded the sacrum area of the back to maintain the comfortability by reducing the area covered by the inductive sensor. Additionally, reducing the placement area of the sensor from L1–S5 to L1–L5 provided a flatter surface, which also avoided the introduction of wrinkles. Figure 9 shows the maximum dimensions of the sensor. These dimensions were used as a reference when designing and evaluating the inductive sensor through theoretical calculations and simulations.

### 3.2. Simulating Inductance Value of a Rectangle Using Ansys

**Figure 10:**
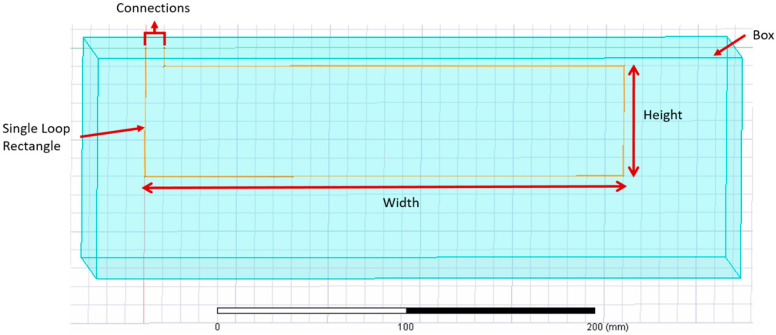
Single loop rectangle simulated in Ansys for Section 3.1 and Section 3.2.

A series of simulations were performed in Ansys 2019 R2/19.4 Electromagnetics (Ansys Inc., Canonsburg, PA, USA) using Ansys Maxwell 3D design. Table 5 shows the parameters used in the Ansys simulations for this section. Figure 10 illustrates the characteristics of the single loop rectangle used for simulations in Section 3.2.1 and Section 3.2.2.

In Table 5, “Sensor’s Characteristics” describes the properties used in this study for all the simulations performed in Ansys. Moreover, Ansys Maxwell 3D required delimitation of the space, denoted by “Box” in Table 5, and to specify the material of the object, which in this case was air.

#### 3.2.1. Inductance Change Based on Perimeter and Area

This section explores the effect of changing the perimeter and area of a single loop rectangular sensor on the inductance value. The performed simulations were divided into two sets, keeping the area constant in one set and keeping the perimeter constant in the other one. Table 6 and Table 7 show the specifications of the first and second set of simulations, respectively.

#### 3.2.2. Inductance Change Based on Height and Width

This section investigates the variations in the inductance of a single loop rectangle with changing the height and width. Similar to the previous section, simulations were divided into two sets, each maintaining either a constant height or a constant width for the single loop rectangle. Table 8 shows the specifications of the first and second sets of simulations.

#### 3.2.3. Inductance Change Based on the Number of Loops in a Flat Rectangular Coil

**Figure 11:**
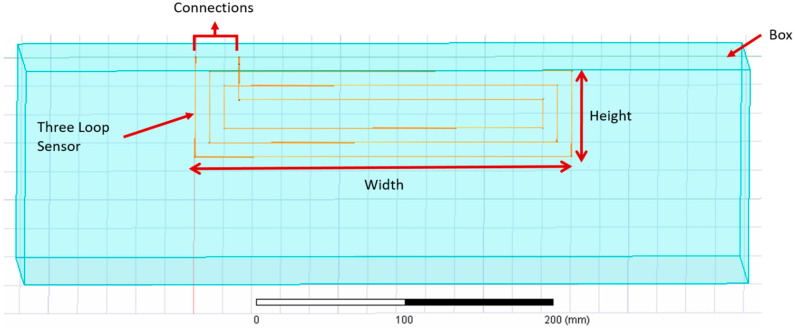
Flat rectangular coil with three turns simulated in Ansys.

The relationship between the inductance value and the number of loops in a flat rectangular coil was also investigated. The distance between each loop *D* was arbitrarily set to 10 mm. Figure 11 illustrates an example of the flat rectangular coil simulated in Ansys.

### 3.3. Results

This section compares the results obtained from theoretical calculations in Section 2.2 and simulations in Section 3.2. The data of both sections were processed using MATLAB R2018b (The MathWorks, Inc., Natick, MA, USA).

#### 3.3.1. Comparison between Calculations and Simulations: Inductance Change Based on Perimeter and Area

Table 9 and Table 10 show the results of the calculated inductance from simulations. In Table 9, the area was kept constant, while in Table 10 the constant parameter was the perimeter.

**Figure 12:**
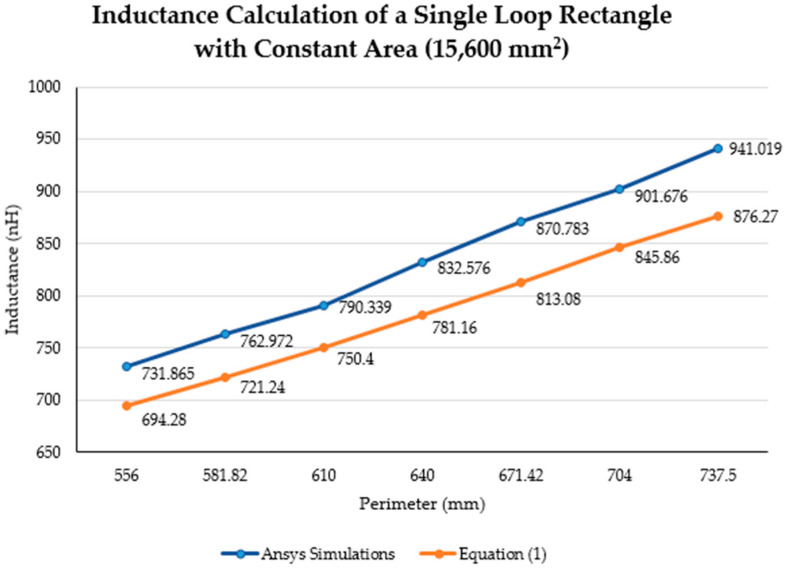
Theoretical and simulated inductance calculation (nH) with a constant area (15,600 mm^2^).

**Figure 13:**
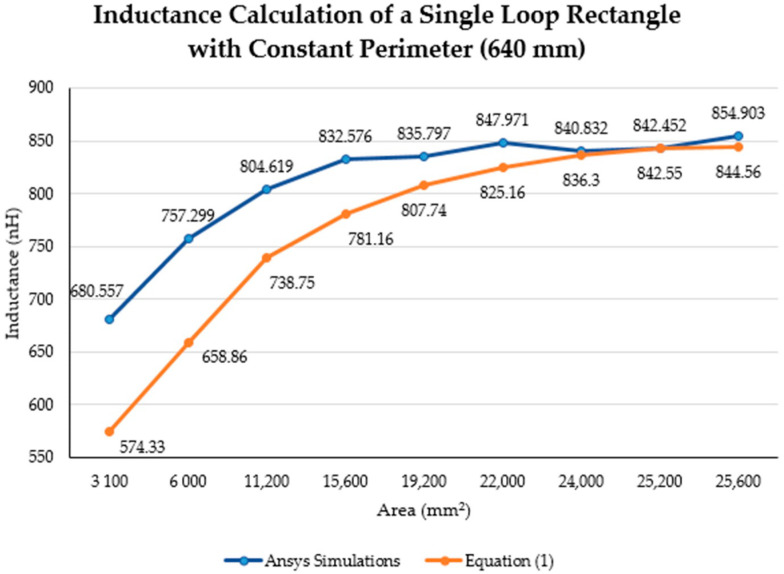
Theoretical and simulated inductance calculation (nH) with a constant perimeter (640 mm).

Figure 12 and Figure 13 illustrate the inductance behavior calculated using Equation (1) (blue curve) as well as the simulation results (orange curve), when the area is constant, respectively.

**Figure 14:**
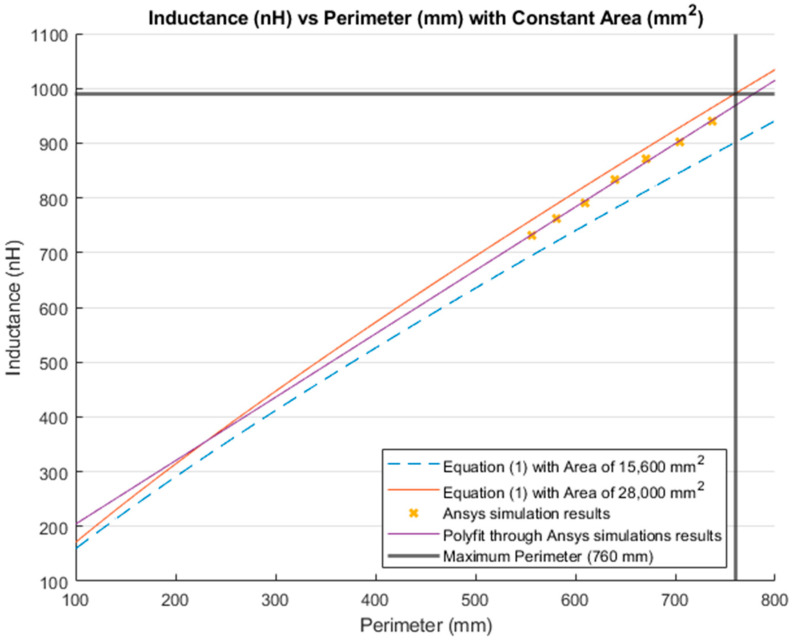
Comparison between the theoretical inductance calculations using Equation (1) with a constant area (mm^2^) and simulations results.

**Figure 15:**
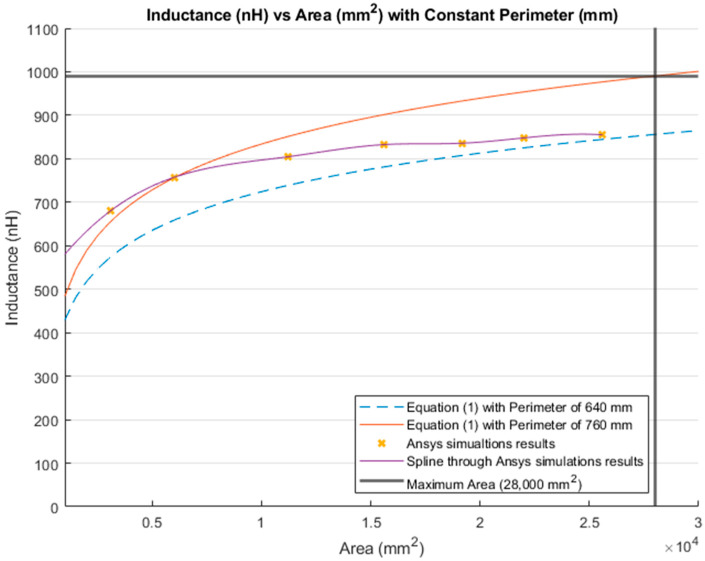
Comparison between theoretical inductance calculations using Equation (1) with a constant perimeter (mm) and simulations results.

Figure 14 and Figure 15 show the comparison between simulation results and theoretical calculations. In Figure 14, the inductance values when the area was kept constant are presented. The blue dashed line is the inductance value that resulted from Equation (1) using a constant area of 15,600 mm^2^; while the red line is the inductance calculated using the same equation, but using the maximum dimensions of the lumbar area (28,000 mm^2^) presented in Section 3.1. Furthermore, the yellow “*x*” represents the inductance resulting from simulations with a constant area of 15,600 mm^2^. The purple line is the MATLAB polynomial curve fitting (polyfit) function using a first-degree polynomial. Finally, the bold grey lines represent the maximum perimeter for the lumbar section of a healthy participant (760 mm).

Figure 15 illustrates the inductance calculations when the perimeter was held constant. Similar to Figure 10, the blue dashed line represents the inductance calculations using Equation (1) with a constant perimeter of 640 mm. The inductance obtained using the same equation with the maximum perimeter of the lumbar section (760 mm) is depicted as a red line. Moreover, the yellow “*x*” represents the inductance obtained by the simulations with a constant perimeter of 640 mm. The purple line is the MATLAB cubic spline data interpolation (spline) function that passes through the simulations results. Finally, the bold grey lines represent the maximum area (28,000 mm^2^) for the lumbar section of a healthy participant.

The inductance value corresponding to the maximum dimensions of the lumbar area of a healthy female participant (280 mm × 100 mm) using Equation (1) was calculated to be 990.41 nH.

#### 3.3.2. Comparison between Calculations and Simulations: Inductance Change Based on Height and Width

Table 11 shows the inductance results from simulations when the height was kept constant. Table 12 presents the inductance obtained from simulations when the width was held constant.

**Figure 16:**
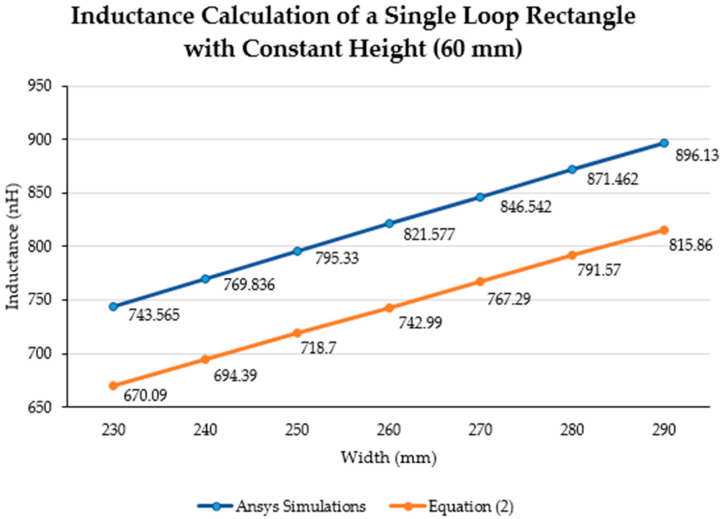
Inductance calculation (nH) using Equation (2) and Ansys simulations with a constant height (60 mm).

**Figure 17:**
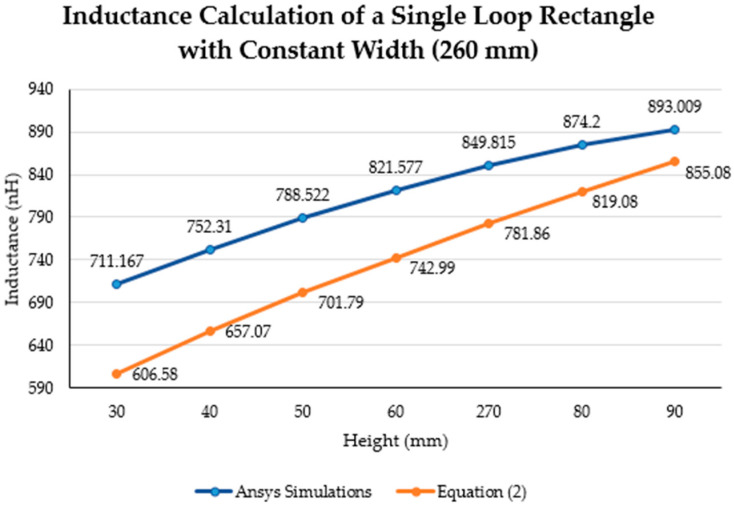
Inductance calculation (nH) using Equation (2) Ansys simulations with a constant width (260 mm).

The inductance behavior with changing the width and height is shown in Figure 16 and Figure 17, respectively.

**Figure 18:**
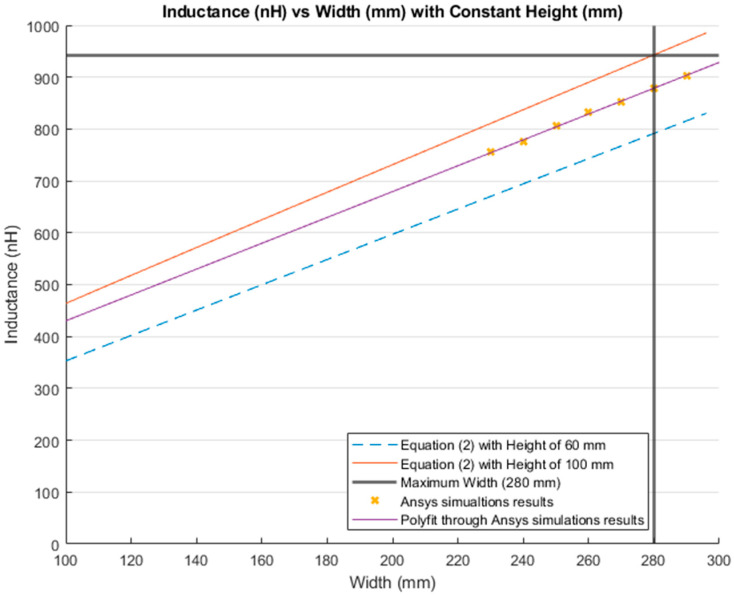
Comparison between theoretical inductance calculations using Equation (2) and simulations results with a constant height (mm).

**Figure 19:**
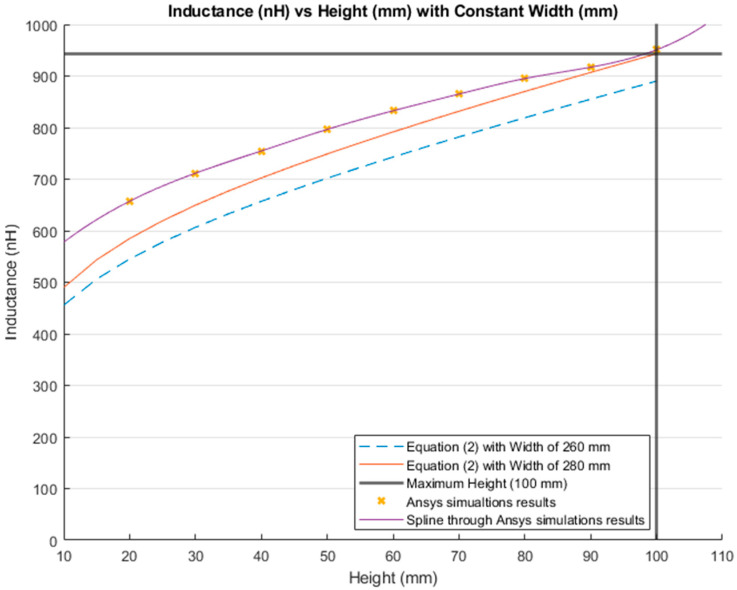
Comparison between the theoretical inductance calculations using Equation (2) and simulation results using a constant width (mm).

Figure 18 and Figure 19 show the comparison between the results of simulations and those of Equation (2). Figure 18 illustrates the inductance values with varying the width and maintaining a constant height. The blue dashed line presents the results from Equation (2) with a constant height of 60 mm. The red line is the inductance calculated from the same equation, but with a constant height of 100 mm; which is the total height of the lumbar section according to the anthropometrics represented in Section 3.1. Additionally, the bold grey lines represent the maximum width for the lumbar section (280 mm). The yellow “*x*” represents the inductance values simulated with a constant height of 60 mm. Finally, the purple line is the MATLAB polynomial curve fitting (polyfit) function using a first-degree polynomial.

Figure 19 shows the results of calculating the inductance value with a variable height and a constant width using Equation (2). The blue and red lines represent the inductance results calculated with a constant width of 260 mm and 280 mm, respectively. The bold grey lines represent the maximum lumbar height (100 mm). The yellow “*x*” markers represent the inductance results from the simulations run using a constant width of 260 mm. Finally, the purple line is the MATLAB cubic spline data interpolation (spline) function based on the simulation results.

The inductance value obtained from the maximum dimensions of the lumbar section (280 mm × 100 mm) using Equation (2) was 943.01 nH.

#### 3.3.3. Comparison between Calculations and Simulations: Inductance Change Based on the Number of Loops in a Flat Rectangular Coil

This section presents the change in the inductance value with varying the number of complete loops using Equation (3). The chosen dimensions for the flat rectangular coil were 60 mm height and 260 mm width. As mentioned in Section 3.2.3, the distance between each loop was 10 mm. The maximum number of complete turns able to fit in the rectangle with the aforementioned dimensions was 3. The MATLAB cubic spline data interpolation (spline) function was used to interpolate the behavior of the results.

**Figure 20:**
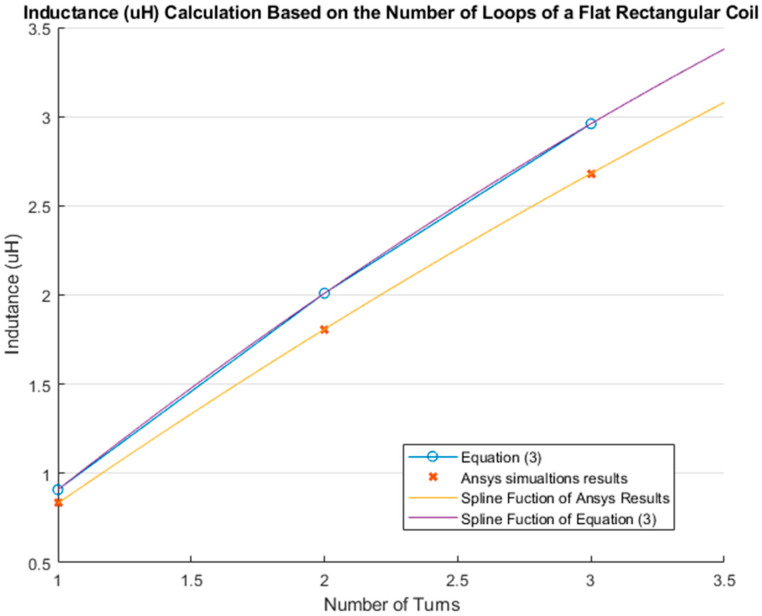
Comparison between the results obtained from Equation (3) and simulations.

Figure 20 illustrates the comparison between the theoretical results from Equation (3) and simulations in which results of Equation (3) results are denoted with a blue “o” markers and simulations results are marked with orange “*x*” markers. The MATLAB cubic spline data interpolation (spline) function was used to extrapolate the values and generate the corresponding curve for each case.

## 4. Discussion

The inductance of a single loop rectangle was calculated using two different equations. Figure 2 shows the results of Equation (1), which describes the inductance based on the perimeter and the area. Equation (1) demonstrated an almost linear behavior when the area was kept constant. However, in the case of a constant perimeter, the inductance behavior was similar to that of a logarithmic graph. Equation (2), which relates the inductance value to the height and width of the rectangle (Figure 3), described the inductance with a linear behavior when the height was constant. On the other hand, when the width was constant, the inductance showed a linear behavior when the height was approximately 25 mm. Unfortunately, these two equations led to different results for the inductance of a single loop rectangle, such that there was a difference of approximately 40 nH between the inductance values calculated using these equations for the same sensor dimensions. This discrepancy in calculated values increased as the rectangle became bigger. An example of this discrepancy can be seen using the lumbar anthropometric dimensions of a healthy participant from Section 3.1, where the rectangle had a width of 280 mm and a height of 100 mm. Using Equation (1), the inductance value was calculated to be 990.42 nH, while using Equation (2), the inductance was equal to 943.01 nH. The difference between these results was 47.41 nH. The equations used in this chapter are solely based on the geometry of the rectangle loop and entirely neglect the material from which the rectangle loop is made.

Finally, when the inductance was studied based on the height and width rather than the area and perimeter, it was possible to observe a more linear behavior, which facilitates the theoretical prediction of the inductance when using a rectangular shape. The inductance calculation based on the area and perimeter reported had closer results to the simulations compared to the results based on width and height. The average difference between the simulation results and Equation (1) calculations was 49.849 nH and 43.066 nH for constant area and constant perimeter, respectively. The average difference between the simulation results and Equation (2) calculations was 77.650 nH and 75.164 nH for a constant height and width, respectively. Additionally, the simulated inductance value using the lumbar anthropometric dimensions was 1.003 µH. The difference between this simulation and the results from Equations (1) and (2) using the same lumbar dimensions was 12.58 nH and 59.99 nH, respectively.

In general, the behavior and trend of inductance values were similar in both simulations and theoretical calculations, but the obtained inductance values were different. Nonetheless, the simulations were closer to the results of Equation (1) than to those of Equation (2). All simulations resulted in a higher inductance compared to theoretical calculations. This outcome could be a result of considering the material of the rectangle loop and the environment surrounding the rectangle loop while running the simulations. Future investigation should include an analysis on the change of the inductance value when a biological body is in close proximity to the inductive textile sensor. Furthermore, unlike studies such as [4,5], the equations presented in this study do not consider mutual-inductance or self-inductance. However, inserting these parameters into the calculations increased the complexity.

The dimensions of the inductive textile sensor were chosen based on using the anthropometric dimensions of the lumbar area of a healthy participant and according to the inductance behavior. A rectangle of smaller dimensions (260 mm width and 60 mm height) was arbitrary selected to compare the inductance value against the maximum inductance for the lumbar section of the back. Based on Equation (1), this smaller rectangle covered up to 78.81% of the maximum inductance range. While using Equation (2), the same smaller rectangle covered up to 78.79%. The maximum inductance was obtained by using the dimensions of the entire lumbar section of a healthy participant, and is presented in Section 3.1.

**Figure 21:**
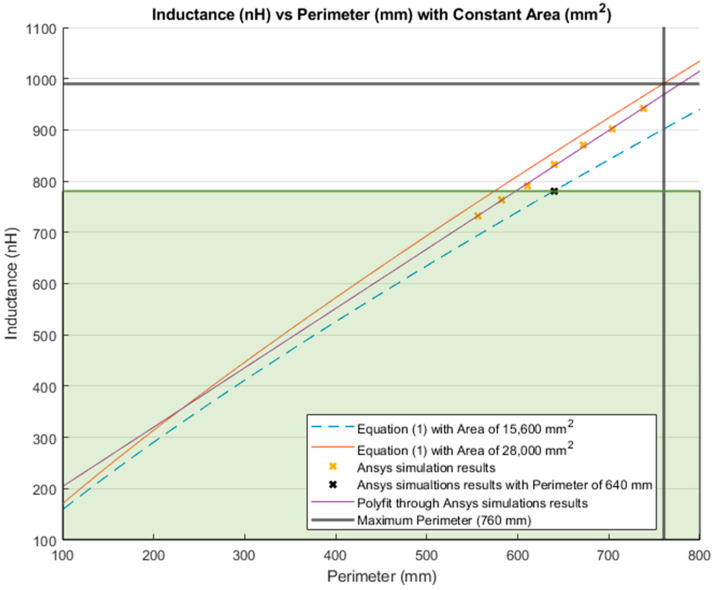
Inductance calculation with a constant area (mm^2^) and a variable perimeter (mm). Highlighted in green shading is 78.81% of the total inductance change.

**Figure 22:**
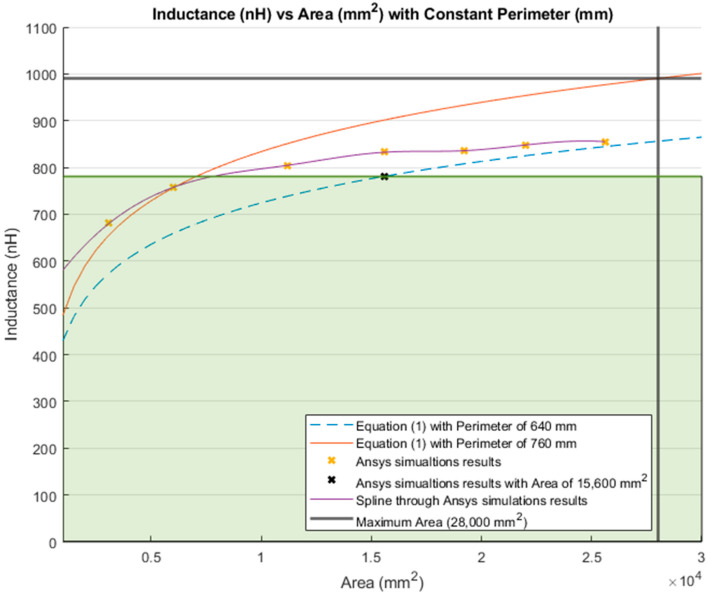
Inductance calculation with a constant perimeter (mm) and a variable area (mm^2^). Highlighted in green shading is 78.81% of the total inductance change.

**Figure 23:**
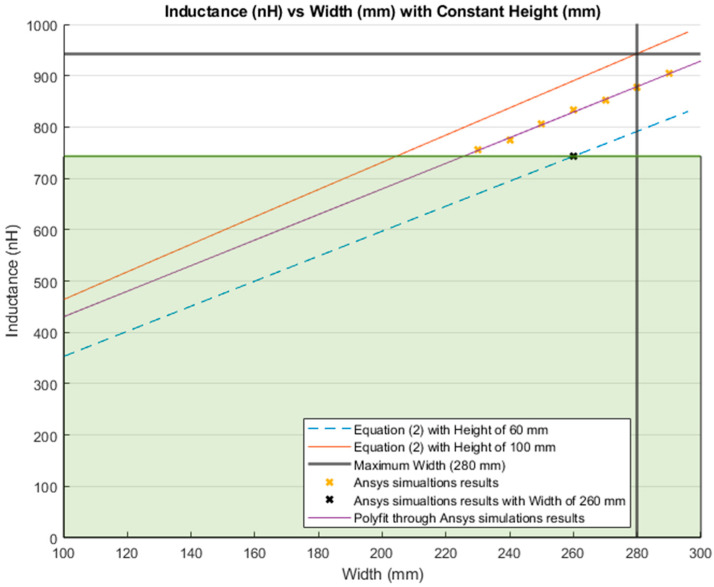
Inductance calculation with a constant height (mm) and a variable width (mm). Highlighted in green shading is 78.79% of the total inductance change.

**Figure 24:**
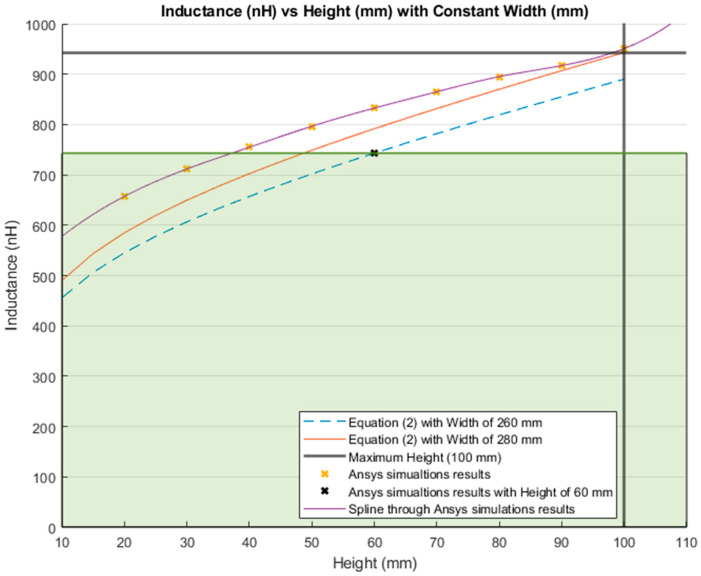
Inductance calculation with a constant width (mm) and a variable height (mm). Highlighted in green shading is 78.79% of the total inductance change.

Figure 21 and Figure 22 show the covered area based on Equation (1). Moreover, Figure 23 and Figure 24 illustrate the covered area based on Equation (2). In both cases, the covered inductance change is highlighted in grey and the black “*x*” represents the simulation results for a rectangle with dimensions 260 mm × 60 mm.

Results of Equation (3) were also different from those of the simulations. The difference between the inductance value calculated with Equation (3) and the simulated one increased with the number of loops. More specifically, the inductance value for a single loop was calculated to be 0.909 µH using Equation (3), while simulations yielded an inductance value of 0.833 µH for the same case, resulting in a difference of 0.0759 µH between the two methods. When considering three loops, the difference in the inductance value increased to 0.279 µH, which was more than twice that obtained for a single loop. Nonetheless, the behavior and trend of inductance were similar in both methods, as shown in Figure 16.

The percentage reduction of the size and inductance value from the total lumbar dimensions to the arbitrary chosen dimension (260 mm width and 60 mm height) were as follows; the area was reduced to 44.29%, the perimeter was reduced to 15.79%, the height was reduced to 66.67%, and finally the width was reduced to 7.14%. These size modifications resulted in a reduction of the inductance by 21.19% and 21.21% for Equations (1) and (2), respectively. Reducing the perimeter and width has a greater impact on the inductance value than on the area and the height. As shown in Figure 22 and Figure 24, the inductance behavior, when modifying the area and height, followed the pattern of a logarithm. Therefore, when deciding the size of the sensor, it was better to modify the area or height to avoid a drastic decrease on its inductance. Additionally, increasing the number of complete loops without modifying the area increased the inductance value.

The theoretical inductance value of a flat rectangular coil of 260 mm width and 60 mm height with three complete loops using Equation (3) was 2.960 µH, while the simulated inductance value was 2.681 µH. The inductance value difference between the simulation and Equation (3) was 0.279 µH. The fabricated inductive strain textile sensor presented in our previous study had a value of 4.500 µH [3]. However, this inductive strain textile sensor was made with a zigzag pattern and included a connection line along the spine. As discussed in Section 2.4: Zigzag properties, use of a zigzag pattern increases the inductance value by 35%. Therefore, the inductance value after increasing the simulated value (2.681 µH) by 35% was 3.619 µH. The difference between the simulation result and the fabricated sensor presented in [3] was 24.309%. This difference was due to the connection line not considered in this study and small differences between the simulation and actual parameters, since the inductive textile sensor was manufactured by hand. Moreover, a simulation presented in our previous study [1], where a simulation of the inductive strain textile sensor included the zigzag pattern and the connection line obtained an inductance value of 4.698 µH. The difference in inductance value between this last simulation and the manufactured inductive textile sensor was 4.4%.

A rectangle of 260 mm width and 60 mm height proved to cover up to 78.8% of the maximum possible inductance value in both Equations (1) and (2), and consequently, was suggested to be an optimal option when the size of the inductive textile sensor was chosen. Additionally, the maximum number of loops that could be fitted into the aforementioned dimensions was three. Considering that the sensor was made of non-stretchable material, increasing the number of loops inevitably increased the stiffness of the fabric, which could interfere with the comfort for the user. Among the important requirements for wearable devices are comfort and being as unobstructive as possible, so users can perform their regular activities as normally as possible [1,6,8,9].

## 5. Conclusions

A design process for an inductive sensor with a flat rectangular coil configuration was presented in this study. The design process was then evaluated by presenting an example case, where the inductive sensor was design to monitor forward bending movements.

The results of the inductive strain textile sensor design and validation presented in this study showed a similar behavior and trend of inductance values in theoretical calculations and simulations, but the obtained values were different. Equation (1) was reported to have closer inductance results to the simulations than Equation (2). The inductance value has a linear behavior using Equations (1) and (2), when the area and height are kept constant, respectively. An inductive sensor of 260 mm width and 60 mm height covered up to 78.8% of the maximum possible inductance value for both equations. The maximum number of loops capable of fit into the aforementioned dimensions was three. Therefore, we considered that the optimal size of the inductive strain textile sensor was a flat rectangle coil of 260 mm width and 60 mm height with three complete loops.

## Figures and Tables

**Table 1:** Correction values of constant *A* in Equation (3) from 0.01 to 0.1.

Wire Diameter/*D*	*A*
0.01	−4.048
0.02	−3.355
0.03	−2.950
0.04	−2.662
0.05	−2.439
0.06	−2.256
0.07	−2.102
0.08	−1.969
0.09	−1.851
0.1	−1.746

**Table 2:** Correction values of constant *B* in Equation (3) from 1 to 10.

Number of Turns (*n*)	*B*
1	0.000
2	0.114
3	0.166
4	0.197
5	0.218
6	0.233
7	0.244
8	0.253
9	0.260
10	0.266

**Table 3:** Ansys parameters used for simulating inductance value for different materials.

Ansys’ Parameters		Sensor
Sensor’s Characteristics	Between Connections	10 mm
	Total Height	60 mm
	Total Length	50 mm
	Material	Copper, Silver, Gold, Stainless steel
	Wire Diameter	0.14 mm
Box Characteristics	X	150 mm
	Y	120 mm
	Z	100 mm
	Material	Air
Setup	Maximum # Passes	10
	% Error	1
	% Refinement Per Pass	30
	Minimum # of Passes	5
	Minimum Converged Passes	1

**Table 4:** Anthropometry dimensions of a healthy female of 25–40 years old.

Trunk’s Anthropometry	
Trunk width at the iliac crest	28 cm
Trunk length C7–L5	41.7 to 42.5 cm
Waist height	103.4 cm
Trochanteric height	82.4 cm

**Table 5:** Parameters used for simulating inductance value using Ansys.

Ansys’ Parameters		Sensor
Sensor’s Characteristics	Material	Copper
	Wire Diameter	0.14 mm
Box Characteristics	X	600 mm
	Y	150 mm
	Z	100 mm
	Material	Air
Setup	Maximum # Passes	10
	% Error	1
	% Refinement Per Pass	30
	Minimum # of Passes	5
	Minimum Converged Passes	1
Mesh	Classic, Small	--
Excitation	--	1.56 mA

**Table 6:** Single loop rectangle dimensions with a constant area (15,600 mm^2^).

Perimeter (mm)	Width (mm)	Height (mm)
556	200	78
581.82	220	70.91
610	240	65
640	260	60
671.42	280	55.71
704	300	52
737.5	320	48.75

**Table 7:** Single loop rectangle dimensions with a constant perimeter (640 mm).

Area (mm^2^)	Width (mm)	Height (mm)
3100	310	10
6000	300	20
11,200	280	40
15,600	260	60
19,200	240	80
22,000	220	100
24,000	200	120
25,200	180	140
25,600	160	160

**Table 8:** Single loop rectangle dimensions with keeping either height or width constant.

Constant Height (60 mm)	Constant Width (260 mm)
Width (mm)	Height (mm)
230	30
240	40
250	50
260	60
270	70
280	80
290	90

**Table 9:** Inductance calculation of a single loop rectangle with a constant area (15,600 mm^2^) using Ansys simulations and Equation (1).

Perimeter (mm)	Width (mm)	Height (mm)	Simulation Inductance (nH)	Equation (1) Inductance (nH)
556	200	78	731.865	694.28
581.82	220	70.91	762.972	721.24
610	240	65	790.339	750.40
640	260	60	832.576	781.16
671.42	280	55.71	870.783	813.08
704	300	52	901.676	845.86
734.5	320	48.75	941.019	876.27

**Table 10:** Inductance calculation of a single loop rectangle with a constant perimeter (640 mm) using Ansys simulations and Equation (1).

Area (mm^2^)	Width (mm)	Height (mm)	Simulation Inductance (nH)	Equation (1) Inductance (nH)
3100	310	10	680.557	574.33
6000	300	20	757.299	658.86
11,200	280	40	804.619	738.75
15,600	260	60	832.576	781.16
19,200	240	80	835.797	807.74
22,000	220	100	847.971	825.16
24,000	200	120	840.832	836.30
25,200	180	140	842.452	842.55
25,600	160	160	854.903	844.56

**Table 11:** Inductance calculation of a single loop rectangle with a constant height (60 mm) using Ansys simulations and Equation (2).

Width (mm)	Simulations Inductance (nH)	Equation (2) Inductance (nH)
230	743.565	670.09
240	769.836	694.39
250	795.330	718.70
260	821.577	742.99
270	846.542	767.29
280	871.462	791.57
290	896.130	815.86

**Table 12:** Inductance calculation of a single loop rectangle with a constant width (260 mm) using Ansys simulations and Equation (2).

Height (mm)	Simulations Inductance (nH)	Equation (2) Inductance (nH)
30	711.167	606.58
40	752.310	657.07
50	788.522	701.79
60	821.577	742.99
70	849.815	781.86
80	874.200	819.08
90	893.009	855.08

## Data Availability

Data sharing not applicable.
